# Three-Dimensional Modeling of Spun-Bonded Nonwoven Meso-Structures

**DOI:** 10.3390/polym15030600

**Published:** 2023-01-24

**Authors:** Zhenxia Ke, Lingjie Yu, Guanlin Wang, Runjun Sun, Mengqiu Zhu, Hanrui Dong, Yiqin Xu, Mengyue Ren, Sida Fu, Chao Zhi

**Affiliations:** 1 School of Textile Science and Engineering, Xi’an Polytechnic University, Xi’an 710048, China; 2 China-Australia Institute for Advanced Materials and Manufacturing, Jiaxing University, Jiaxing 314001, China; 3 Key Laboratory of Functional Textile Material and Product, Xi’an Polytechnic University, Ministry of Education, Xi’an 710048, China

**Keywords:** spun-bonded nonwoven fabric, image processing, 3D model, finite element method

## Abstract

As a type of fiber system, nonwoven fabric is ideal for solid–liquid separation and air filtration. With the wide application of nonwoven filter materials, it is crucial to explore the complex relationship between its meso structure and filtration performance. In this paper, we proposed a novel method for constructing the real meso-structure of spun-bonded nonwoven fabric using computer image processing technology based on the idea of a “point-line-body”. Furthermore, the finite element method was adopted to predict filtration efficiencies based on the built 3D model. To verify the effectiveness of the constructed meso-structure and simulation model, filtration experiments were carried out on the fabric samples under different pollution particle sizes and inlet velocities. The experimental results show that the trends observed in the simulation results are consistent with those of the experimental results, with a relative error smaller than 10% for any individual datum.

## 1. Introduction

Research studies show that the morphology of nonwoven filter material has an important influence on its filtration performance [1,2]. Therefore, it is of great significance to measure the morphological parameters of fiber in nonwoven filter material to clarify the relationship between the structure and filtration performance and realize structural optimization and performance improvements.

Currently, the finite element method (FEM) has been widely used to predict the mechanical and filtration properties of fiber filtration materials. Elnaz Saberi et al. [3] simulated the puncture behavior of nonwoven meso-structures with finite element methods based on the hyperelastic model, and they observed that the nonwoven fabric with higher weights showed higher puncture stress. Artem Kulachenko et al. [4] established a three-dimensional random fiber mesh finite element model using deposition technologies and simulated the deformation and failure behavior of the fiber mesh. Alvaro Ridruejo et al. [5] used the finite element method to simulate the mechanical behavior of glass fiber felt before fracture. N Chen et al. [6] modeled the three-dimensional geometric model of polypropylene nonwoven meso-structures and predicted the bond performance of the model using finite element simulation. Hou X.N et al. [7] established a discontinuous finite element model, studied the deformation mechanism of nonwoven samples in two main directions, and numerically analyzed the influence of discontinuous and nonuniform fiber mesh and different joint point arrangements. In finite element analysis, the effectiveness of the simulation results heavily depends on the established meso-structure model. The model that is more similar to the real internal structure of the nonwoven filter material is higher in reference value. Therefore, a real 3D model of nonwoven filter materials is rather crucial.

However, due to the complex internal structure and disordered spatial arrangement of fibers within nonwoven fabrics, accurately extracting each fiber’s morphology for modeling is difficult. Optical microscopy is one of the most important methods in fiber detection because it can measure fiber morphological characteristics quickly and accurately without introducing damage at a certain magnification [8,9]. Image processing technology based on an optical microscope is very important for establishing a 3D model of nonwoven fabric. Jing et al. [10] calculated fiber orientations and length distributions by using the 3D model of a fiber net of wool nonwoven material established by micro-CT. Huang et al. [11] explored the 3D skeleton image technology for establishing metal fiber mesh and calculated the morphological parameters such as the curvature and orientation of fiber fragments using skeleton tracking. Yu et al. [12] obtained a microscopic image of a continuous section of the prefabricated fiber net of the needled carbon-carbon composite by using a layer removal method, and they established a 3D microscopic finite element model after image segmentation and noise reduction. Although the above method retains the real shape data of fiber in the nonwoven material fiber net, it can obtain an accurate model and has great development potential [12]. However, due to the large fiber slenderness ratio and fiber bending, fibers within nonwoven fabrics inevitably involve lamination, interlacing, and entanglement. Therefore, the following two problems will be caused in the image acquisition process. Firstly, the three-dimensional stacking of fibers will cause the depth of the nonwoven fabric to exceed the microscopic system’s depth of field, which is the so-called “multi-focal plane” phenomenon [13]. Secondly, due to interlacing and entanglement among fibers, some fibers parts are blocked, which causes difficulty in accurately extracting their complete morphology, thus reducing the accuracy of subsequent image detection results [11].

The reported studies show that image fusion technology can effectively expand the depth of field of the microscopic system [14,15]. Image fusion utilizes the focusing layers of different depths, which can solve the “multi-focal plane” problem. However, the obtained 3D image still exhibits the occlusion of targets since depth information is still lost [16]. Therefore, to establish the real 3D meso-structure of nonwoven fabrics, the problems overlapping the occlusion of the fiber system need to be completely solved.

**Figure 1:**
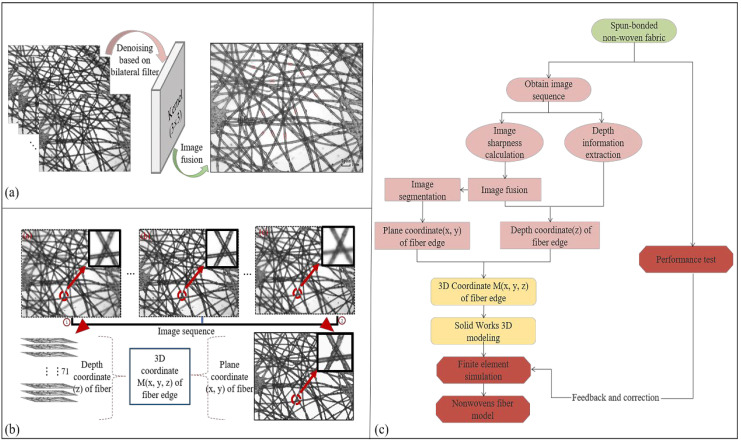
Process of image fusion and 3D coordinate extraction. (**a**) Image sequence fusion; (**b**) spatial 3D coordinate acquisition; (**c**) experimental steps.

The optical microscope cannot directly obtain the depth information of the target. Some scholars collect multiple sequence images by controlling the continuous movement of the object platform in the vertical direction and reconstructing the 3D surface of the target by focusing on the depth information [17,18,19]. Based on this idea, this paper explores the method of reconstructing the surface depth image of the nonwoven fabric, thus extracting 3D fiber information. Due to the mutual occlusion of fibers, an optical microscope is limited to acquiring surface depth. It cannot directly obtain the spatial morphology and three-dimensional coordinates of the shielded part of fibers. Therefore, a “point-line-volume” modeling method based on 2D sequence images is proposed in this paper. A 3D model similar to the real meso-structure of nonwoven filter materials is established based on a series of 2D images (as shown in Figure 1c).

This work aims to develop an effective method to establish the real meso-structure model of nonwoven fabric, thereby predicting the filtration performance with the help of FEM. The processing steps of sequential images are discussed in Section 2. Then, the meso-structure of the nonwoven fabric is reconstructed in Section 3. Section 4 introduces research on finite element simulations. In Section 5, the filtration experiments were carried out. Finally, the conclusions were obtained in Section 6.

## 2. Image Processing Method

The sample used in this paper comprises a piece of polypropylene spun-bond nonwoven fabric with a weight of 18 g/m^3^ and a thickness of 0.08 mm. Seventy-one images were collected by an optical microscope under different focal positions. Parts of the captured images are shown in Figure 1a.

### 2.1. Image Processing

Before the image fusion process, a bilateral filtering model was adopted to denoise the captured images.

The bilateral filter is a nonlinear filter with edge-preserving denoising properties. The bilateral filter optimizes the weighted coefficient of the Gaussian filter and convolves with the gray information of the image’s pixels. In the bilateral filter, the pixel values in the region near the pixel point are weighted by a certain combination. The calculation formula is shown in Equation (1):

(1) $$\left\{\begin{array}{c} g(i,j)=\frac{\sum_{kl}f(k,l)u(i,j,k,l)}{\sum_{kl}u(i,j,k,l)}\\ d(i,j,k,l)=\exp(-\frac{{\left(i-k\right)}^{2}+{\left(j-l\right)}^{2}}{2\sigma_{d}^{2}})\\ r(i,j,k,l)=\exp(-\frac{f(i,j)-f{\left(k,l\right)}^{2}}{2\sigma_{r}^{2}})\\ u(i,j,k,l)=\exp(-\frac{{\left(i-k\right)}^{2}+{\left(j-l\right)}^{2}}{2\sigma_{d}^{2}}-\frac{f(i,j)-f{\left(k,l\right)}^{2}}{2\sigma_{r}^{2}}) \end{array}\right.$$

Here, (*k*, *l*) represents the central coordinates of the filter window, and (*i*, *j*) indicates the coordinates of other coefficients of the filter window. *f* (*i*, *j*) denotes the pixel value of the image at the point (*i*, *j*), and *f* (*k*, *l*) is the pixel value of the point (*k*, *l*); *σ* is the standard deviation of the Gaussian function. The weighted coefficient *u* (*i*, *j*, *k*, *l*) is the product of proximity factor *d* and brightness factor similarity factor *r* in pixel space.

### 2.2. Image Sharpness Evaluation

Since the spun-bonded nonwoven images selected in this paper have rich details and edge information, to obtain the 3D coordinate information of fibers in the nonwoven fiber, a sharpness evaluation algorithm a regional gradient variance algorithm is adopted [20].

The algorithm uses a 7 × 7 sharpness window to evaluate the sharpness of the central pixel centering upon the pixel for which its sharpness needs to be measured, which can be expressed as Equation (2):

(2) $$V_{n}(i,j)=\sum \left(T_{n}(a,b)-U_{n}{\left(i,j\right)}^{2}\right)$$

where *T_n_* (*a*, *b*) represents the gradient of pixels (*a*, *b*) in the 7 × 7 sharpness window, *U_n_* (*i*, *j*) represents the average gradient of all pixels in the 7 × 7 sharpness window, and *V_n_* (*i*, *j*) represents the sharpness of the center pixel of the 7 × 7 sharpness window in the *i*th focused image layer.

Gradient *T_n_* (*a*, *b*) is calculated by the Prewitt operator, and the vertical and horizontal directions of the Prewitt operator are first-order differential operators, as shown in Equation (3):

(3) $$T_{n}(a,b)=abs(f(a,b)⊕\left|a^{\prime }b^{\prime }c^{\prime }\right|)+abs(f(a,b)⊕\left|d^{\prime }d^{\prime }d^{\prime }\right|)$$

where |*a*′ *b*′ *c*′| and |*d*′ *d*′ *d*′| are Prewitt operators, and ⊕ represents convolution calculations.

### 2.3. Image Fusion

In the image sequence, the focus degree of the single fiber in different spatial positions is different, so extracting the single fiber in one captured image layer is difficult. Therefore, it is necessary to fuse the image sequence to obtain a full clear image of the single fiber.

For sample images, the sharpness of each pixel point in each focused image is calculated using Equation (4) and recorded in the 3D coordinate matrix *M*(*i*, *j*, *n*), where *i* is the image’s pixel width, *j* is the image’s height, and *n* is the number of the image. This method compares all image frames (*n* = 1, 2, …, 71) in the same position (*i*, *j*), calculates the focus image frame *k* with the highest resolution (*i*, *j*), which is denoted as *H*(*i*, *j*, *k*), and stores (*i*, *j*, *k*) coordinate information (*x*_1_, *y*_1_, *z*_1_), as shown in Equation (4):

(4) $$H(i,j,k)=\underset{n}{\underset{︸}{\arg\max}}M(i,j,n)$$

As shown in Figure 1a, the fiber focus degree in different image sequences varies, and the fibers interlock with one another, making it difficult for a single fiber to be completely segmented. The multi-focus image fusion algorithm adopted in this paper is the multi-wavelet algorithm. This algorithm carries out a fusion method for high-frequency components based on regional direction and energy weighting, and the fusion rules are described in Equation (5):

(5) $$G_{j.D}^{\gamma }=W_{j.\max}^{\gamma }G_{j.A}^{\gamma }+W_{j.\min}^{\gamma }G_{j.B}^{\gamma }$$

In the formula, $W_{j.\max}^{\gamma }$ and $W_{j.\min}^{\gamma }$ correspond to the maximum and minimum values of the energy matching degree, respectively. γ=1,2,3 corresponds to the three directions of 0, 45, and 90. $G_{j.A}^{\gamma }$ and $G_{j.B}^{\gamma }$ correspond to the j-grade decomposition coefficients of the original image A and B, respectively. $G_{j.D}^{\gamma }$ is the final fuse image.

### 2.4. Fiber Fineness Measurement

In nonwovens, fibers are overlapped and distributed randomly in three dimensions, which makes it difficult to measure the fiber’s diameter in nonwovens materials. This paper uses digital image processing technology to measure fiber diameter in fiber mesh. The fused image in Figure 1a shows all fibers in the net mesh. The pixel scale in the figure is 0.01 mm for every 7 pixels. Ten fiber segments were randomly selected from the fusion image to calculate the diameter of a single fiber, and diameter lines were drawn across the parallel edges of each fiber segment. The fiber diameter was obtained by measuring the diameter line of each fiber segment and taking the average value. The calculation is shown in Equation (6):

(6) $$d=\frac{N}{\varepsilon }\times 0.01$$

where *N* represents the number of pixels and *ε* is the pixel scale.

The diameter of the fiber in the spun-bonded nonwoven fabric is randomly distributed. To reduce the calculation’s cost, the diameters of all fibers within one nonwoven fabric are assumed to be constant and thus set as *d*, as obtained from Equation (6). Finally, we calculated that the diameter of the fibers in the spun-bonded nonwovens is 0.025 mm. Figure 1 shows the image fusion process and 3D coordinate extraction.

## 3. Three-Dimensional Model Reconstruction

### 3.1. Single Fiber Segmentation and 3D Point Cloud Extraction

**Figure 2:**
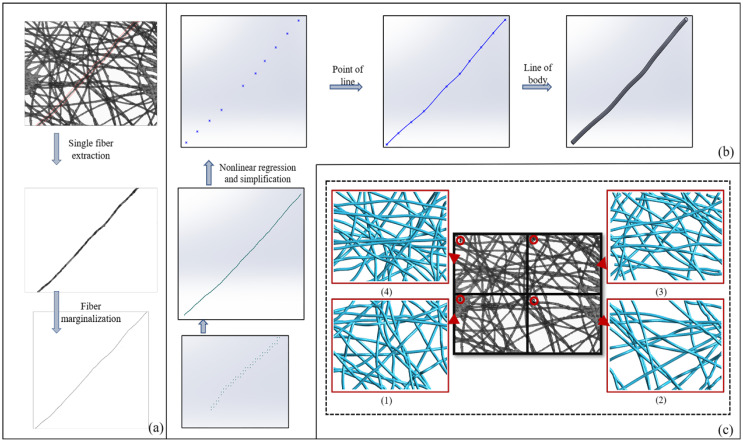
Modeling steps. (**a**) Single fiber extraction and canny edge detection; (**b**) “point-line-body” modeling; (**c**) 3D model.

The entanglement between fibers blocks part of the fibers, so it is impossible to extract its complete morphology. As shown in Figure 2a, for every fiber, several representative dots were selected and recorded along the fiber’s edge before fitting them into a complete fiber curve.

A 3D modeling method of the “point-line-body” is proposed in this paper (Figure 2b). The canny edge detection algorithm is adopted to obtain the fiber’s edge in this paper, as shown in Figure 2a. After recording the plane coordinate (*x*_2_, *y*_2_) of a certain edge dot, the 3D coordinate (*x*_1_, *y*_1_, *z*_1_) can be found by searching the 3D coordinate matrix *M* (*i*, *j*, *n*). The specific steps are shown in Figure 1b.

### 3.2. Three-Dimensional Model Construction

To ensure the 3D model is close to the real spatial structure of the nonwoven fabric, nonlinear regression was used to fit the curve function before establishing the model. This paper selected the Polyfit function [21] to carry out linear fitting and regression analysis, which is calculated by Equation (7):

(7) $$\left[\begin{array}{cccc} x_{1}^{n} & x_{1}^{n-1} & \cdots & 1\\ x_{2}^{n} & x_{2}^{n-1} & \cdots & 1\\ \vdots & \vdots & \ddots & \vdots \\ x_{m}^{n} & x_{m}^{n-1} & \cdots & 1 \end{array}\right]\left[\begin{array}{c} p_{1}\\ p_{2}\\ \vdots \\ p_{n+1} \end{array}\right]=\left[\begin{array}{c} y_{1}\\ y_{2}\\ \vdots \\ y_{m} \end{array}\right]$$

where Polyfit is solved by *p* = *V*/*y*. Nonlinear fitting was carried out by the Polyfit (*x*, *y*, *n*) function, and three-dimensional spatial coordinates were simplified, as shown in Figure 2b. The final three-dimensional model is shown in Figure 2c.

## 4. CFD Simulation Research

### 4.1. Model Meshing and Conditional Setting

**Figure 3:**
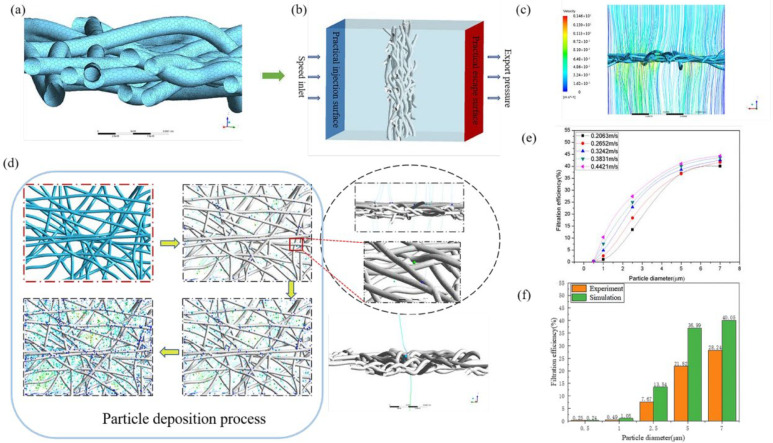
Simulation process. (**a**) Model meshing; (**b**) boundary condition setting; (**c**) trajectories of particles in the model; (**d**) particle deposition process and motion tracking; (**e**) filtration efficiency diagram of the model at different inlet velocities; (**f**) comparison diagram of experimental data and simulation data when the inlet velocity is 0.2063.

This paper used computational fluid dynamics to analyze the 3D model and establish the numerical model. Before the built 3D model is imported into Ansys Fluent for numerical simulation, the 3D model needs to be meshed. The ICEMCFD components were chosen to be contained in the Ansys workbench for 3D model mesh processing. Grid processing was selected to discretize the area of the simulation calculation, which is also the basic link of numerical simulation calculations. The local mesh is shown in Figure 3a.

When the meshed files were imported into Fluent for numerical analysis, the boundary conditions of the calculation region needed to be set, as shown in Figure 3b. In the CFD-coupled calculation, the airflow inlet adopted the velocity-inlet condition, and the outlet adopted the pressure-outlet condition. In the text, the particle size was set between 0.5 μm and 0.7 μm. To ensure the stability of airflow entering the computational domain, the distance between the model’s particle injection surface and the upper surface was set to 2, which is the same as the distance between the particle’s escape surface and the lower surface. When the gas phase calculation reached a stable state, the particles are injected into the calculation domain along with the airflow’s direction. If the particles moved beyond the calculation area or encountered the boundary of the calculation domain. It would be recorded as an escape. Figure 3c shows the particle trajectory in the model.

### 4.2. Governing Equation

The fluid control equation follows the law of mass conservation and energy conservation of the local mean variable. Gidaspow et al. [22]. proposed two models, A and B, which are suitable for the CFD-DEM simulation of gas-solid two-phase flows. Model A assumes that the pressure drop of the flow is borne by both the solid and gas phases, while model B assumes that the pressure drop is borne only by the gas phase. Since the gas passing through the fibrous medium exhibits laminar flows, model A with laminar flow is selected in this paper to solve the equation, and the continuity equation and momentum equation are expressed as Equations (8) and (9):

(8) $$\frac{\partial \varepsilon }{\partial t}+\nabla \cdot (\varepsilon u)=0$$

(9) $$\frac{\partial }{\partial t}(\rho_{f}\varepsilon u)+\nabla \cdot (\rho_{f}\varepsilon uu)=-\varepsilon \nabla P-F_{fp}+\nabla \cdot (\varepsilon \tau )+\rho_{f}\varepsilon g$$

where *ε*, *u*, *ρ_f_*, *P*, and *τ* represent the tensors of porosity, average fluid velocity, three-dimensional density, pressure, and viscous fluid stress, respectively. *F_fp_* is the total interaction forces between particles in grid cells, including the object resistance, pressure gradient force, etc.

(10) $$F_{fp}=\frac{\sum f_{d,i}}{\Delta V\cdot n}$$

where *f_d,i_* is the force acting on particles, and ∆*V* and *n* denote the volume of grid computing cells and the number of particles within the volume of each grid cell.

In the process of particle filtering, particles and fibers produce an interaction between particles and gases so that momentum and energy exchange occur between the particles. This paper studies the contact model used in the particle: The solid nonlinear softball model. In the solid two-phase flow, according to Newton’s second law, the translation and rotation motion equation of the particle can be obtained, and the expression is as follows:

(11) $$m_{p}\frac{dU_{p.i}}{dt}=F_{fp,i}^{\prime }+F_{nc,i}+f_{n,i}+f_{,i}$$

(12) $$I_{p}\frac{dw_{i}}{dt}=T_{i}$$

where *m_p_*, *U_p,i_*, *f_n,i_*, and *T_i_* represent the mass, velocity and angular velocity, tangential impact force, and the impact moment of the particulate matter, respectively. *I_p_* denotes the inertial moment of the particle, and *F*′*_fp,i_* and *F_nc,i_* denote fluid and non-contact forces.

To improve the accuracy of the numerical simulation results, the SIMPLE algorithm was selected in Ansys Fluent to solve the coupling process between the velocity and pressure fields.

### 4.3. Numerical Simulation

The simulated particles move through the 3D model under the action flow field and pressure in the numerical simulation process. The simulation calculation will judge whether the particles interact with the fiber or are captured by the fiber. In this paper, a Lagrange multiphase discrete phase model (DPM) in Fluent was selected to track and solve the motion trajectory of the particles.

First, the material property of particles is set as anthracite particles, the velocity inlet of the fluid is set as the injection surface of the particles, and the diameter of the particles is set as five gradients: 0.5 μm, 1 μm, 2.5 μm, 5 μm, and 7 μm. The velocity of the inlet is set according to the flow of fluid, and it is divided into five gradients: 0.2063 m/s, 0.2652 m/s, 0.3242 m/s, 0.3831 m/s, and 0.4421 m/s. The collision between the emitted particles and the fibers in the 3D model is considered when the quantitative particles are launched at the same inlet velocity. The difference between particles at the launch inlet and particles escaping from the pressure outlet is the particle numbers captured by the fibers; the particles touching the boundary of the computational domain are also regarded as an escape. During the simulation calculation, if the distance between the particle and the fiber surface in the 3D model is less than or equal to the particle’s size, the particle is considered to be captured by the fiber.

Figure 3d shows the deposition process of the particles on the fiber. The movement track of the particles can be observed from the side view. It can be observed from the detailed figure on the upper right that the particle’s sizes vary. Particles with larger particle sizes are easily captured by fibers, while particles with smaller particle sizes easily escape. In addition, the motion of individual particles is shown on the lower right side of the graph. Figure 3e shows the movement track of the particle in the model. It can be observed that the direction changes during the moving process in the flow field, which is more consistent with the movement law of the particle in the actual filtration experiment.

Figure 3b shows the filtration efficiency of the model for particles with 0.5 μm, 1 μm, 2.5 μm, 5 μm, and 7 μm particle sizes at the inlet velocity of 0.2063 m/s, 0.2652 m/s, 0.3242 m/s, 0.3831 m/s, and 0.4421 m/s. The filtering efficiency, *η*, is defined as follows:

(13) $$\eta =\frac{C_{\mathrm{out}}-C_{\mathrm{in}}}{C_{\mathrm{out}}}\times 100\%$$

where *C*_out_ represents the number of particles before filtering, and *C*_in_ represents the number of particles after filtering.

## 5. Results and Discussion

### 5.1. Filtration Experiments

**Figure 4:**
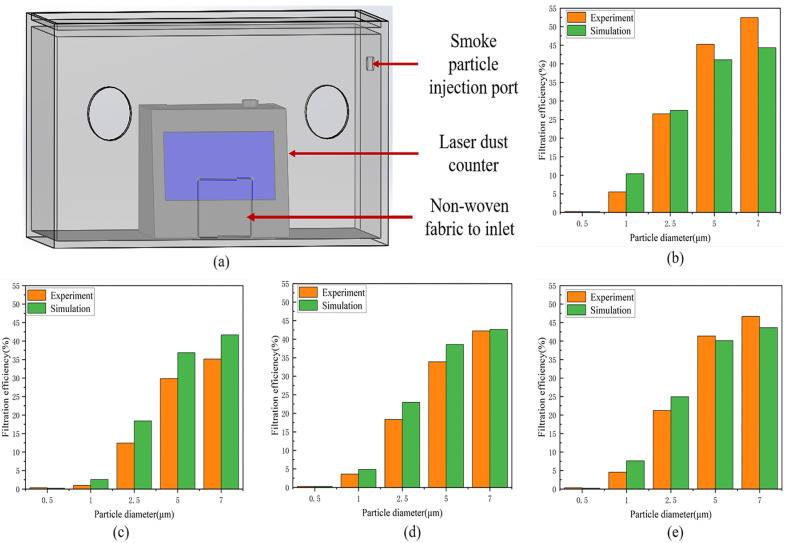
(**a**) Experimental device diagram; (**b**–**e**) are the schematic diagrams of the comparison between experimental and simulated data when the inlet velocity is 0.2652 m/s, 0.3642 m/s, 0.3831 m/s, and 0.4421 m/s, respectively.

In this paper, a laser dust particle counter (LX-600S) was used to measure the filtering efficiency of nonwoven fabric samples with particle sizes of 0.5 μm, 1 μm, 2.5 μm, 5 μm, and 7 μm particles at gas flow rates of 0.2063 m/s, 0.2652 m/s, 0.3242 m/s, 0.3831 m/s, and 0.4421 m/s. A certain amount of smoke particles will be injected into the sandbox to ensure enough particulate matter with a particle size of 2.5, 5, and 7 in the sandbox, as shown in Figure 4a.

### 5.2. Data Analysis

Figure 3e shows the filtration efficiency of the model for particles with particle sizes of 0.5 μm,1 μm, 2.5 μm, 5 μm, and 7 μm at different velocity inlets. It was observed that the filtration efficiency of particles with the same particle size increases with the increase in the number of fibers in the model under the same inlet velocity. The filtration efficiency of the model increases with the increase in particle size. In the same model, the filtration efficiency of particles with the same particle size increases with the increase in inlet velocity. At the same time, with the increase in spun-bonded nonwoven meso-fabric weight, the filtering efficiency of spun-bonded nonwoven fabric increases.

As shown in Figure 3f and Figure 4b–e, the filtration efficiency of finite element simulation was compared with that of the experiment. The trends in the simulation’s results are consistent with that of the experimental results. With the increase in inlet velocity, the filtration efficiency of particles with the same particle size increased. At the same inlet velocity, the filtration efficiency in particles increases with the increase in particle size. Therefore, the model established in this paper is proven to be effective.

To verify the influence of different fabric densities on filtration efficiency, we conducted experiments on three different nonwovens samples. It can be observed from Table 1 that the filtration efficiency increases with the increase in fabric density. In addition, to verify the model’s validity, we calculated the errors between the simulation data and experimental data. It can be observed from Table 1 that the errors between the simulated and the experimental data are all within 10%. The *error* is calculated in Equation (14):

(14) $$error=\frac{x_{1}-x_{2}}{x_{1}}\times 100\%$$

where *x*_1_ represents the larger experimental data and simulation data, and *x*_2_ indicates the smaller experimental data and simulation data.

## 6. Conclusions

This paper proposed a “point-line-body” 3D modeling method for spun-bonded nonwoven fabrics based on a series of captured 2D images. First, optical microscopy was used to capture the image sequence of nonwoven fabrics. Multi-focus image fusion was used to fuse the image and extract the 3D coordinates of each fiber. Subsequently, SolidWorks was used to establish a 3D model of spun-bonded nonwoven fabrics. Finally, the finite element method was adopted to simulate nonwoven materials’ filter performance. The simulation results show that the influence trends of the fiber number, particle size, and inlet velocity on the filtration efficiency of spun-bonded nonwoven fabrics in this model are the same as the experimental results of real nonwoven fabrics. This indicated that the proposed method is effective for 3D modeling spun-bonded nonwoven fabrics.

## Figures and Tables

**Table 1:** Comparison experiment and simulation of three different samples.

Sample	Weight/g	Thickness/mm	Particle Size/μm	Experiment/%	Simulation/%	Error/%
1	9	0.11	0.3	60.05	64.93	7.5
			0.5	68.19	72.84	6.3
			1.0	88.71	85.93	3.1
			3.0	93.01	95.38	2.5
			5.0	100	98.39	1.6
2	15	0.16	0.3	78.49	85.24	7.9
			0.5	84.61	91.73	7.8
			1.0	96.11	95.36	0.8
			3.0	98.19	99.12	0.9
			5.0	100	100	0
3	28 g	0.28	0.3	88.64	92.21	3.9
			0.5	93.05	94.85	1.8
			1.0	98.35	96.83	1.6
			3.0	99.79	99.57	0.2
			5.0	100	100	0

## Data Availability

The data used to support the findings of this study are available from the corresponding author upon request.

## References

[1] Pradhan A.K., Das D. (2018). A comparative study on filtration performance of mono-, bi-, and multi-constituent nonwoven air filter media. J. Text. Inst..

[2] Liu X., Shen H., Nie X. (2019). Study on the filtration performance of the baghouse filters for ultra-low emission as a function of filter pore size and fiber diameter. Int. J. Environ. Res. Public Health.

[3] Saberi E., Najar S.S., Abdellahi S.B., Soltanzadeh Z. (2017). A hyperelastic approach for finite element modelling puncture resistance of needle punched nonwoven geotextiles. Fibers Polym..

[4] Kulachenko A., Uesaka T. (2012). Direct simulations of fiber network deformation and failure. Mech. Mater..

[5] Ridruejo A., González C., Llorca J. (2010). Damage micromechanisms and notch sensitivity of glass-fiber nonwoven felts: An experimental and numerical study. J. Mech. Phys. Solids.

[6] Chen N., Silberstein M.N. (2017). Determination of bond strengths in nonwoven fabrics: A combined experimental and computational approach. Exp. Mech..

[7] Hou X., Acar M., Silberschmidt V.V. (2010). Non-uniformity of deformation in low-density thermally point bonded nonwoven material: Effect of microstructure. J. Mater. Sci..

[8] Wu Y., Li D., Li Z., Yang W. (2014). Fast processing of foreign fiber images by image blocking. Inf. Process. Agric..

[9] Li S. (2012). Measurement of diameter and scale of cashmere fibers by computer images analysis. J. Fiber Bioeng. Inform..

[10] Jing H., Yu W. (2018). Estimation of fiber orientation and length distribution in cashmere fibrous assemblies. Text. Res. J..

[11] Huang X., Wen D., Zhao Y., Wang Q., Zhou W., Deng D. (2016). Skeleton-based tracing of curved fibers from 3D X-ray microtomographic imaging. Results Phys..

[12] Yu J., Zhou C., Zhang H. (2017). A micro-image based reconstructed finite element model of needle-punched C/C composite. Compos. Sci. Technol..

[13] Liu Y., Liu S., Wang Z. (2015). Multi-focus image fusion with dense SIFT. Inf. Fusion.

[14] Tian J., Chen L. (2012). Adaptive multi-focus image fusion using a wavelet-based statistical sharpness measure. Signal Process..

[15] Huang W., Jing Z. (2007). Multi-focus image fusion using pulse coupled neural network. Pattern Recognit. Lett..

[16] Wang R., Xu B., Li C. (2013). Accurate fiber orientation measurements in nonwovens using a multi-focus image fusion technique. Text. Res. J..

[17] Abrahamsson S., Chen J., Hajj B., Stallinga S., Katsov A.Y., Wisniewski J., Gustafsson M.G. (2013). Fast multicolor 3D imaging using aberration-corrected multifocus microscopy. Nat. Methods.

[18] Moeller M., Benning M., Schonlieb C., Cremers D. (2015). Variational depth from focus reconstruction. IEEE Trans. Image Process..

[19] Fujii H., Kodama K., Hamamoto T. Scene flow estimation through 3D analysis of multi-focus images. Proceedings of the 2016 Visual Communications and Image Processing (VCIP).

[20] Yu L., Wang G., Zhi C., Xu B. (2019). 3D web reconstruction of a fibrous filter using sequential multi-focus images. Comput. Model. Eng. Sci..

[21] Liu C., Cheng I., Basu A., Ye J. (2017). Robust MRI abnormality detection using background noise removal with polyfit surface evolution. EURASIP J. Image Video Process..

[22] Gidaspow D., Jung J., Singh R.K. (2004). Hydrodynamics of fluidization using kinetic theory: An emerging paradigm. Powder Technol..

